# The effectivity of the new improved Maastricht brace compared with the Boston brace

**DOI:** 10.1186/1748-7161-7-S1-O41

**Published:** 2012-01-27

**Authors:** J Hermus, M Hulsbosch, C Arts, L Van Rhijn

**Affiliations:** 1 Maastricht University Medical Center, Maastricht, The Netherlands

## Background

The brace 2000 was developed to improve the compliance of brace treatment and therefore its results. We further developed the brace 2000 in the Maastricht brace (M brace) to enhance the effectivity of the brace and its wearing comfort.

## Materials and methods

Pressure measurements were performed in 4 patients wearing the Boston brace and the M brace to understand the efficacy between the two braces without intervariability [1,2].

## Results

The mean primary right thoracic curve was 29° uncorrected; the mean secondary curve measured 19°. In the Boston brace group the mean primary right thoracic curve was 22° ; the mean secondary curve measured 16°. The mean corrective force over the lumbar brace pad in standing position was 382 N; over the thoracic brace pad it was 285 N. In the M brace group the mean primary right thoracic curve was 20° ; the mean secondary curve measured 13°. The mean corrective force over the lumbar brace pad in standing position was 373 N; over the thoracic brace pad it was 311 N. Difference in M-brace and Boston brace was not significant, for the lumbar brace pad: 0.12, for the thoracic brace pad 0.07 with ANOVA analysis.

## Conclusions

There is a tendency that the M Brace give a higher pressure comparing to the classic Boston brace with a better correction on the spinal radiograph. Bracing results are directly related to compliance with brace treatment; therefore, optimal results cannot be achieved without the patient's cooperation and family support. Brace mechnisms can be explained and understood by giving more insight about pressure/force measurements in the brace. The Quality of life measured with the SRS 22 and Brace questionnaire was higher in the Brace 2000 than the Boston brace.
